# Effect of a Synbiotic Containing *Lactobacillus paracasei* and *Opuntia humifusa* on a Murine Model of Irritable Bowel Syndrome

**DOI:** 10.3390/nu12103205

**Published:** 2020-10-20

**Authors:** Gyeol Seong, Seungbaek Lee, Yang Won Min, Yeon Sil Jang, So-Young Park, Cheol-Hyun Kim, Chansu Lee, Sung Noh Hong, Dong Kyung Chang

**Affiliations:** 1 Department of Medicine, Samsung Medical Center, Sungkyunkwan University School of Medicine, Seoul 06351, Korea; gyeol.seong@gmail.com (G.S.); seungbaek12@gmail.com (S.L.); ys.jang@skku.edu (Y.S.J.); cslhero@gmail.com (C.L.); gisnhong@gmail.com (S.N.H.); do.chang@samsung.com (D.K.C.); 2 Laboratory of Pharmacognosy, College of Pharmacy, Dankook University, Cheonan 31116, Korea; soypark23@dankook.ac.kr; 3 College of Biotechnology & Bioengineering, Dankook University, Cheonan 31116, Korea; hichkim@dankook.ac.kr

**Keywords:** irritable bowel syndrome, probiotics, prebiotics, synbiotics, *Lactobacillus paracasei*, *Opuntia*

## Abstract

The administration of a combination of probiotics and prebiotics is expected to be a promising strategy for improving irritable bowel syndrome (IBS) symptoms. This study aimed to investigate the efficacy of a synbiotic containing *Lactobacillus paracasei* and *Opuntia humifusa* extract for symptomatic improvement of IBS in a murine model and to evaluate the mechanism underlying the beneficial effects of this synbiotic. A total of 20 male Wistar rats aged 8 weeks with IBS induced by restraint stress were assigned into four groups and administered *L. paracasei* as a probiotic and *O. humifusa* extract as a prebiotic for 4 weeks. The primary outcome was stool consistency at week 4. To evaluate the mechanism underlying the beneficial effects of the synbiotic, fecal microbial analysis was conducted, and the serum corticosterone levels, tumor necrosis factor-α (TNF-α) levels in the colon tissue, and expression of tight junction proteins were investigated. All three treatment groups showed significantly lower scores for stool consistency than the control group at week 4 (all *p* < 0.001). When compared with the control group, the synbiotic groups showed a significantly greater abundance of *L. paracasei* in fecal microbial analysis, lower serum corticosterone levels, lower TNF-α levels in the colon tissue, and higher expression of tight junction proteins. This novel synbiotic containing *L. paracasei* and *O. humifusa* extract can improve the stool consistency in a murine model of IBS. It may be a promising treatment option for IBS, and human studies are warranted.

## 1. Introduction

Irritable bowel syndrome (IBS) is a chronic functional gastrointestinal (GI) disorder characterized by recurrent abdominal pain associated with defecation and change in the frequency or form of stools [1,2]. As the pathophysiology of IBS has not been fully understood, the treatment strategies for IBS target the predominant symptoms. Recent studies have attempted to provide a better understanding of the pathophysiology by presenting new disease concepts of IBS, including disorder of the brain-gut axis, altered gut microbiota [3], cytokine response [4], low-grade mucosal inflammation [5], and abnormal change in the intestinal permeability [6].

Similar to that in other GI disorders, altered gut microbiota is also associated with the symptoms and severity of IBS [7,8,9]. In this regard, modulating the gut microbiota may improve the IBS symptoms; therefore, a broad spectrum of probiotics and prebiotics have been studied. A probiotic is a living organism beneficial to the host [10], and a prebiotic is a non-viable food component associated with the regulation of microbiota [11,12]. If prebiotics provide synergistic benefits in combination with probiotics, the combination is termed synbiotic. The administration of probiotics and prebiotics is a potential treatment option for IBS, based on the available evidence that suggests that probiotics and prebiotics exercise anti-inflammatory and immunomodulatory effects on the intestinal mucosa [13,14]. Some specific bacteria, including *Lactobacillus* and *Bifidobacterium,* are widely known to aid in improving the IBS symptoms [15].

*Lactobacillus* provides effective symptom relief in IBS [16]. *L. paracasei* DKGF 1 is a strain extracted from Kimchi, a traditional Korean lactic acid-fermented food. Previous studies have reported that it can reduce the stress-induced fecal output in IBS [17,18,19]. Genus *Opuntia,* commonly called prickly pear, is a member of the plant family Cactaceae. Isorhamnetin, a flavonoid from *Opuntia humifusa,* was reported to be a scavenger of free radicals and modulates the expression of inflammatory cytokines; thus, isorhamnetin possesses potential anti-oxidative and anti-inflammatory activities [20,21]. Moreover, dietary fibers from *Opuntia*, composed of mucilage and pectin, may be taken up by lactic acid-producing bacteria; the production of short-chain fatty acids by lactic acid-producing bacteria may accordingly increase, leading to the induction of the prebiotic effects of these bacteria [22,23]. In the light of these data, we expected extracts from *Opuntia* to show a synbiotic effect on the survival and beneficial activity of *L. paracasei*, leading to symptomatic improvement in IBS. Therefore, in this study, we aimed to assess the effect of a synbiotic containing *L. paracasei* and *O. humifusa* on improving the symptoms in a murine model of IBS and to investigate the mechanism of action of the synbiotic.

## 2. Methods

### 2.1. Bacterial Strains and Materials

We used *L. paracasei* DKGF 1 as a probiotic, and *O. humifusa* extract as a prebiotic. *L. paracasei* DKGF 1 was isolated from Kimchi, cultivated and maintained in MRS (Man, Rogosa, and Sharpe; Difco Laboratories, Detroit, MI, USA) at 37 °C [24,25]. *O. humifusa*, locally called as Cheonnyuncho, is widely cultivated in the southern coastal regions of South Korea. The harvested stems of *Opuntia* were washed, freeze-dried, and grounded and were considered raw material. After repeated extraction performed three times for 3 h at 100 °C in hot water, the prebiotic samples were obtained. *Opuntia* extracts were filtered using filter paper and then concentrated at 40 °C in a rotary vacuum evaporator (Eyela, Tokyo, Japan). Maltodextrin, probiotics, and prebiotics were obtained from GF FERMENTECH (GF FERMENTECH Ltd., Chungcheong, Korea) and the samples were prepared by dissolving in distilled water.

### 2.2. Rats and Study Design

**Figure 1:**
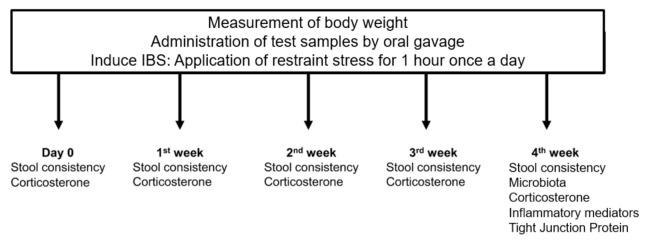
Study design.

Male Wistar rats (weight: 350 ± 50 g) aged 8 weeks (Oriental Bio Co, Seongnam, Korea) were maintained in a sterile pathogen-free environment. The animals were single-housed in plastic cages, under a 12 h light: 12 h dark cycle at constant temperature and humidity; they had *ad libitum* access to food and water. After 1 week of acclimatization, the rats were randomly divided into the following groups with 5 rats in each group: (1) Control (Maltodextrin); (2) Treatment group 1 [Maltodextrin + *L. paracasei* (1 × 10^10^ cfu/g)]; (3) Treatment group 2 [Maltodextrin + *L. paracasei* (1 × 10^10^ cfu/g) + *Opuntia* extract (10.0 mg%, *w*/*w*)]; (4) Treatment group 3 [Maltodextrin + *L. paracasei* (1 × 10^10^ cfu/g) + *Opuntia* extract (30.0 mg%, *w*/*w*)] [26,27]. They were orally administered the test sample, depending on their group, for 4 weeks. To develop an IBS model where IBS was induced by chronic restraint stress, all groups were immobilized using plastic restrainers, allowing a close fit for 1 h daily during the 4 weeks of treatment (Figure 1) [28].

During the study period, the bodyweight of the rats was checked daily, and stool consistency and serum corticosterone levels were assessed on the last day of every week. The primary outcome was stool consistency. We assessed the stool consistency using a three-grade score (0, normal; 2, loose; and 4, diarrhea) in a blinded manner. Fecal microbiota, inflammatory cytokine levels in the colon tissue, and expression of tight junction proteins were investigated at the end of the experimental period.

The study protocol was approved by the Institutional Animal Care and Use Committee of Samsung Biomedical Research Institute (Seoul, Korea. IRB No. 20170216002). As an accredited facility of the Association for Assessment and Accreditation of Laboratory Animal Care International (AAALAC International), the SBRI abides by the Institute of Laboratory Animal Resources (ILAR) guide.

### 2.3. Fecal Samples and Microbial Analysis

The fecal samples were collected on the last day of the 4th week and stored at −80 °C until genomic DNA (gDNA) extraction. DNA was extracted using FastDNA SPIN Kit for Soil (no. 116560200; MP Biomedicals, Solon, OH, USA). The DNA sequence was analyzed using Illumina MiSeq platform (Illumina, San Diego, CA, USA) by ChunLab Inc. (Seoul, Korea). All raw reads were checked quality, and low quality (<Q25) reads were filtered using the Trimmomatic 0.321 software [29]. The pair-end sequence (250 base pair) data were merged using PANDASeq2 software. Mothur’s pre-clustering program was used to merge the resulting sequences and to extract unique sequences, allowing up to two differences between them. It helped to remove noise from the sequences. Of all the sequences that passed these processes, 20,000 reads were randomly selected and confirmed as chimera reads with a best-hit similarity rate below 97% using UCHIME algorithm [30]. We analyzed taxonomic profiling using the 16S rRNA database in the EzBioCloud [31]. Sequences with less than a 97% best-hit similarity rate were detected using 16S rRNA database. Statistical testing was analyzed using Mann-Whitney U test. A *p*-value of less than 0.05 was considered statistically significant.

Chao, ACE, and Jackknife method were used to assess species richness. Shannon, NPShannon, Simpson indices, and Phylogenetic diversity were used to assess within-community diversity. The between-sample diversity was calculated using unweighted UniFrac metrics. The β diversity was visualized by hierarchical cluster trees using the unweighted pair group method with arithmetic mean (UPGMA) and principal coordinate analysis (PCoA).

### 2.4. Serum Corticosterone

The serum corticosterone level was measured in the blood samples on the last day of every week. Approximately 500 μL of whole blood was collected in 1.5-mL microcentrifuge tubes containing 10 μL of EDTA (ethylenediaminetetraacetic acid). The serum was obtained by centrifugation for 10 min in a refrigerated centrifuge at 4 °C and stored at −80 °C for further analysis [32,33]. The serum corticosterone level was quantified using a commercially available corticosterone ELISA kit (Arigo, ARG80652, Hsinchu, Taiwan, China), following the manufacturer’s instructions. The absorbance was measured spectrophotometrically at 450 nm using a microtiter plate reader (Varioskan Flash Thermo Scientific, Waltham, MA, USA). The minimal detectable concentration of corticosterone was approximately 6.1 ng/mL [34]. All samples were tested in duplicate.

### 2.5. Assessment of Inflammatory Mediators

Th levels of inflammatory mediators, including interleukin (IL)-1β/IL-1F2, IL-6, IL-10, interferon (IFN)-γ, and tumor necrosis factor-α (TNF-α), were measured in the colon tissue using the Rat premixed Multi-Analyte kit (R&D Systems Europe, Ltd. Abingdon, UK) The reagents and standards were prepared in accordance with the instructions from the manufacturer. The plates were analyzed on a calibrated Luminex^®^ 100/200 reader using MasterPlex QT2010 (MiraiBio, Hitachi, CA, USA). The concentrations were determined from the plate readings and expressed as pg/mL (Supplementary Table S1) [35].

### 2.6. Tight Junction Proteins

The expression of Claudin-1 and zona occludens-1 (ZO-1), types of tight junction proteins in the intestine, were measured using immunohistochemistry (IHC) [36]. The intestinal tissue was sliced into 4-µm thick sections, and they were deparaffinized in xylene, rehydrated in graded alcohol, and transferred to 0.01 M phosphate-buffered saline (PBS; pH 7.4). We used 1× citrate buffer (pH 6.0; Dako, Carpinteria, CA, USA) to perform heat-induced epitope retrieval. The sections were treated with serum-free blocking agent (Dako) to block nonspecific binding for 3 min at 121 °C. To reveal the hidden antigen epitopes, endogenous peroxidase was blocked with 3% hydrogen peroxide in PBS for 10 min at room temperature. After washing with PBS buffer, the sections were treated with a serum-free blocking solution (Dako) for 20 min at room temperature to block nonspecific binding. Subsequently, the sections were incubated overnight at 4 °C with primary antibodies [1:100 or 1:500 rabbit polyclonal anti-claudin-1 (ab140349) or rabbit monoclonal anti-ZO-1 (ab221547)], and then, incubated for 30 min at room temperature with horseradish peroxidase P-labelled polymer conjugated secondary antibodies against mouse IgG (Dako) or rabbit IgG (Dako). The complex was visualized using diaminobenzidine (DAB). The nuclei were lightly stained with Mayer’s hematoxylin [37,38]. We used Image J program to scan the stained slides. The ratio of the positive stained area to the area of the entire scanned specimen was expressed as a percentage.

### 2.7. Statistical Analysis

The data are expressed as mean ± standard error of the mean (SEM) and were analyzed using GraphPad Prism 5 software (GraphPad, San Diego, CA, USA). The body weight was analyzed using two-way analysis of variance (ANOVA) followed by the Bonferroni post-hoc test. One-way ANOVA followed by Tukey post-hoc tests were used to analyze stool consistency, serum corticosterone levels, and inflammatory mediator levels and tight junction protein expression in the colon tissue. *p*-value < 0.05 was considered significant. The significant differences in alpha diversity were calculated using the Wilcoxon rank-sum test. Beta diversity was calculated using unweighted UniFrac metrics.

## 3. Results

### 3.1. Analysis of Body Weight

**Figure 2:**
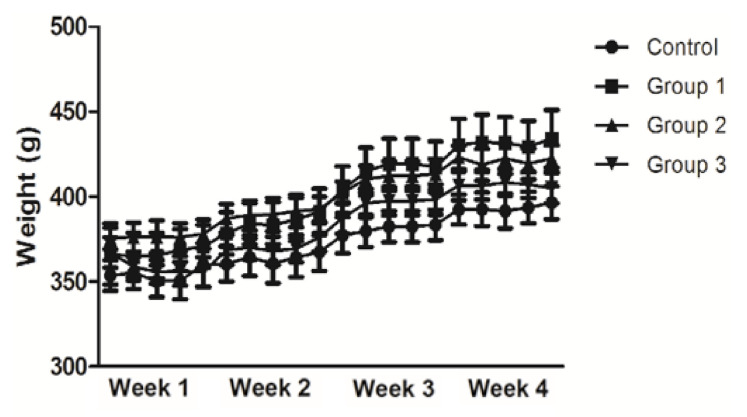
Analysis of body weight Results are expressed as the mean ± SEM (*n* = 5/group). Significant differences were not observed in the body weight among the four groups, as determined using two-way ANOVA with post-hoc Bonferroni correction. ANOVA, analysis of variance; SEM, standard error of the mean.

The body weight increased steadily over the experimental period in all groups (Figure 2). All samples administered orally did not make any significant differences between the groups. At the end of the experimental period, an approximately 1.2% increase in the bodyweight of the rats in all groups was noted (Control: 396.4 ± 9.7 g; Group 1: 434.0 ± 16.9 g; Group 2: 422.4 ± 7.9 g; and Group 3: 405.0 ± 8.9 g, *p* = NS).

### 3.2. Stool Consistency

**Figure 3:**
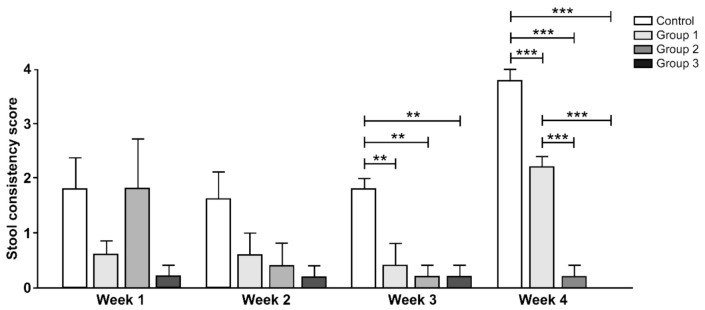
Stool consistency scores. Results are expressed as the mean ± SEM (*n* = 5/group). * indicates significant differences, as determined using one-way ANOVA and Tukey’s test; ** *p*-value < 0.01, *** *p*-value < 0.001. ANOVA: analysis of variance; SEM: standard error of the mean.

The stool consistency score was lower in all treatment groups than in the control group at week 4 (control: 3.8; group 1: 2.2; group 2: 0.2; group 3: 0, all *p* < 0.001). The synbiotic groups (treatment group 2 and 3) showed lower score than treatment group 1 (group 1: 2.2; group 2: 0.2; group 3: 0, *p* < 0.001; Figure 3). In addition, all treatment groups showed significantly lower scores than the control group at week 3 (control: 1.8; group 1: 0.4; group 2: 0.2; group 3: 0.2, all *p* < 0.01). Treatement group 3 showed consistently lower scores than the control group (Supplementary Figure S1). These findings showed the efficacy of *L. paracasei* for improving stool consistency; the findings also indicated that *Opuntia* extract can enhance this effect of *L. paracasei*.

### 3.3. Fecal Microbial Analysis

**Figure 4:**
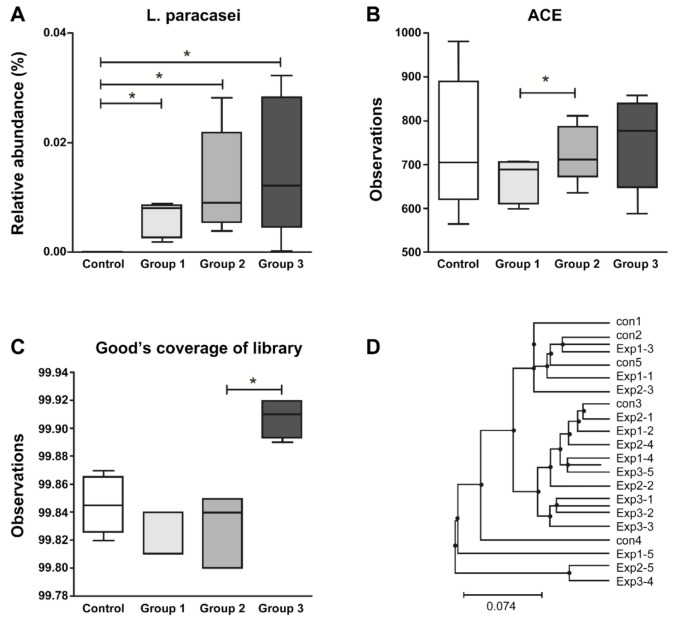
Fecal microbial analysis. Relative abundance of *L. paracasei* (**A**) and alpha-diversity presented by ACE (**B**) and Good’s coverage of library (**C**). UPGMA clustering based on the unweighted UniFrac distance (**D**). The box indicates the interquartile range (IQR), and the mean value is shown as a line within the box; the whiskers extend to the most extreme value within 1.5 × IQR, and the outliers are shown as crosses. * indicates significant differences, as determined using the Wilcoxon rank-sum test; * *p*-value < 0.05. ACE, abundance-based coverage estimator; UPGMA, unweighted pair group method with arithmetic mean.

The relative abundance of *L. paracasei* in the fecal samples was found to be progressively higher in the treatment groups than in the control group (*p* < 0.05; Figure 4). However, a significant difference was not noted between the treatment groups and the control group with respect to alpha and beta diversity (Supplementary Figure S2). Taxonomic relative abundance of bacterial taxa is shown in the Supplementary Table S2.

### 3.4. Serum Corticosterone Levels

**Figure 5:**
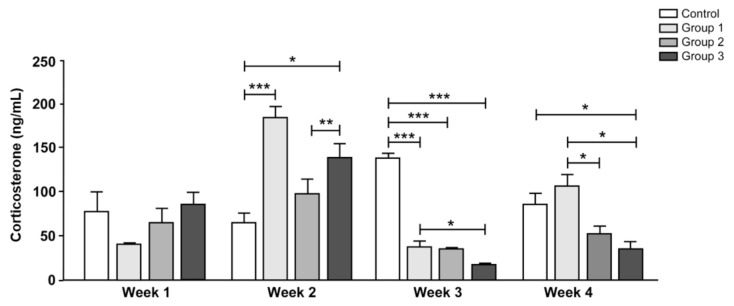
Serum levels of corticosterone. The corticosterone level was measured every week after application of restraint stress for 1 h. Results are expressed as the mean ± SEM (*n* = 5/group). * indicates significant differences, as determined using one-way ANOVA and Tukey’s test; * *p*-value < 0.05, ** *p*-value < 0.01, *** *p*-value < 0.001. ANOVA, analysis of variance; SEM, standard error of the mean.

At week 4, treatment group 3 showed significantly lower serum corticosterone levels than the control group (control: 88.9 ng/mL; group 3: 35.5 ng/mL, *p* < 0.05; Figure 5). Treatment groups 2 and 3 that received the synbiotic also showed lower serum corticosterone levels than treatment group 1 that received only the probiotic (group 1: 108.3 ng/mL; group 2: 55.0 ng/mL; group 3: 35.5 ng/mL, *p* < 0.05). At week 3, the serum corticosterone level was lower in all treatment groups than in the control group (control: 141.0 ng/mL; group 1: 40.2 ng/mL; group 2: 37.3 ng/mL; group 3: 19.9 ng/mL, *p* < 0.001). However, the serum corticosterone level was not lower in the treatment groups compared with that in the control group until week 2.

### 3.5. Inflammatory Mediator Levels

**Figure 6:**
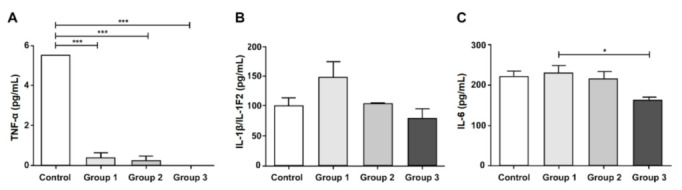
Analysis of the inflammatory mediators. The cytokine levels in the colonic tissue were evaluated, including TNF-α (**A**); IL-1β/1F2 (**B**); IL-6 (**C**). Results are expressed as the mean ± SEM (*n* = 5/group). * indicates significant differences, as determined using one-way ANOVA and Tukey’s test; * *p*-value < 0.05, *** *p*-value < 0.001. ANOVA, analysis of variance; IL, interleukin; SEM, standard error of the mean; TNF-α, tumor necrosis factor-α.

All treatment groups showed lower TNF-α levels in the colonic tissue than in the control group (all *p* < 0.001; Figure 6A). However, there was no difference between the treatment and control groups with respect to the IL-1β/IL-1F2 and IL-6 levels (Figure 6B,C). IL-10 and IFN-γ were not detected in the present study.

### 3.6. Expression of the Tight Junction Proteins in the Colon Tissue

**Figure 7:**
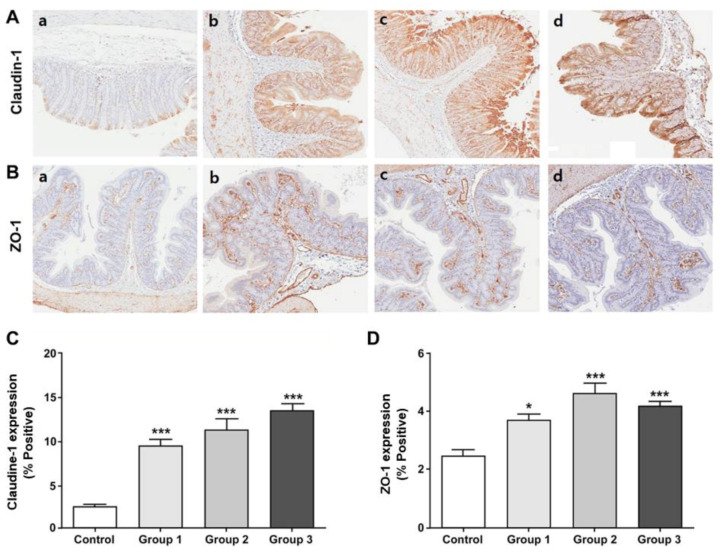
Analyses of the expression of tight junction proteins. Representative H&E-stained images showing (**A**) Claudin-1 and (**B**) ZO-1 expression in the colon obtained during immunohistochemical analysis (magnification, 200×; **a** Control; **b** Treatment group 1; **c** Treatment group 2; **d** Treatment group 3); (**C**) Claudin-1 protein expression and (**D**) ZO-1 protein expression. The ratio of the positive stained area to the entire specimen was expressed as a percentage. Results are expressed as mean ± SEM (*n* = 5/group). * indicates significant differences relative to the controls, as determined using one-way ANOVA and Tukey’s test; * *p*-value < 0.05, *** *p*-value < 0.001. ANOVA, analysis of variance; H&E, hematoxylin and eosin; SEM, standard error of the mean; ZO-1, zona occludens-1.

To identify the association between the stress response of the gut mucosal, we investigated the expression of Claudin-1 and ZO-1 in the colonic tissue. The expression of Claudin-1 was significantly higher in all three treatment groups than in the control group (*p* < 0.001; Figure 7A,C). In addition, ZO-1 showed higher expression in all treatment groups than in the control group (*p* < 0.001; Figure 7B,D).

## 4. Discussion

In this study, we observed that all treatment groups showed better stool consistency than the control group at week 4. In addition, the stool consistency of the synbiotic groups was better than that of the probiotics only group. We also identified the mechanisms underlying the beneficial effect of the synbiotic.

Although several studies have reported on the efficacy of probiotics for the alleviation of IBS symptoms to date, the evidence is still limited and the results are not consistent [39]. These inconsistencies may be due to the heterogeneity in the study design or the limitations associated with the probiotics themselves. As a probiotic is a living organism, it is difficult to ingest adequate amounts of viable bacteria or manufacture probiotic-based products with respect to the technical aspects, costs, and storage issues associated with probiotics. Moreover, issues of quality control, including the selection of the bacterial strain, formulation, safety, and viability in the gut environment, must be addressed for the marketed products. As the use of prebiotics can aid in overcoming these limitations and can enhance the beneficial effects of probiotics with safety and relatively low costs, the use of synbiotics is a cost-effective and promising treatment option for IBS. The positive effects of prebiotics, mostly composed of fibers, on gut microbiota and hosts have been reported in previous studies [40,41,42]. Among many sources of prebiotics, *Opuntia* is well-known for its biological activity as an antioxidant and for its anti-inflammatory effects [43]. Dietary fibers from *O. ficus indica* containing mucilage and pectin may be taken up and fermented by the gut microbiota, thus, inducing the prebiotic effects [22]. While there is substantial evidence supporting the beneficial effects of probiotics and prebiotics on the improvement of IBS, the detailed synergetic effects and mechanism of each combination of probiotics and prebiotics have not been elucidated to date. Our study reported the effect of a synbiotic containing *L. paracasei* and *O. humifusa* on the improvement of stool consistency; additionally, we also provided scientific evidence of the mechanism underlying the beneficial effects of the synbiotic using IBS-related factors reported in previous studies, including the decrease in the serum corticosterone levels, low levels of TNF-α in the colonic mucosa, and increase in the expression of the tight junction proteins. 

The fecal microbial analysis revealed that the relative abundance of *L. paracasei* was significantly higher in the groups treated with the synbiotic compared to that in the control group. One of the most important requirements of a probiotic product is to ensure that a sufficient number of live bacteria reach the intestine [18,44]. For bacteria to reach the intestine alive, the bacteria have to overcome barriers, including low pH, bile, and proteolytic enzymes. *Opuntia*-derived fibers induce the growth up of lactobacilli and bifidobacteria, suppress the growth of enterococci, enterobacteria, staphylococci, and clostria, and increase short-chain fatty acid (SCFA) production in the intestine [22]. The pectin component is tolerable to gastric acid and improves the survival of *Lactobacillus* when used in microencapsulation [44]. Therefore, we expected that extracts from *Opuntia* can induce the growth and survival of probiotic bacteria, leading to the prebiotic effect.

Corticosterone is a major stress-related hormone linked with the hypothalamic-pituitary-adrenal axis (HPA), the core stress response system. IBS is a functional GI disorder associated with both physical and psychological stress [45]. Patients with IBS present with overactivation of the HPA axis and increased inflammatory cytokine levels, which have been implicated in the pathophysiology of IBS [46]. IBS has recently been conceptualized as a disorder of the brain-gut axis. Corticosterone can serve as a link between the brain and intestinal immune system by inducing a decrease in the expression of tight junction proteins in the colonic mucosa and an increase in the mucosal permeability in response to stress [47]. Moreover, recent studies supported that low-grade mucosal inflammation and altered gut permeability may be associated with the development of IBS symptoms, particularly diarrhea-type IBS [48]. The serum TNF-α and IL-6 concentration were higher in patients with IBS-D than in the healthy controls, and the anxiety scores were associated with a high concentration of TNF-α [49]. Furthermore, the expression of the tight junction proteins in the colonic mucosa, such as Claudin-1 and ZO-1, was lower in patients with IBS-D [50]. These changes in the immune response may be associated with alterations in the gut barrier function and visceral hypersensitivity. Patients with post-infectious IBS, who present with an impaired gut barrier and increased intestinal permeability, showed increased stool frequency [51]. In this study, the synbiotic groups showed significantly lower serum corticosterone and TNF-α levels and higher expression of Claudin-1 and ZO-1 at week 4 than the control group. Taken together, our synbiotics may suppress the stress-induced release of serum corticosterone and TNF-α and increase the expression of the tight junction proteins in the colonic mucosa, eventually leading to the maintenance of mucosal integrity and improvement in the stool consistency. Therefore, the use of synbiotics may be an effective option for improving IBS symptoms.

This study used the wrap restraint model introduced by Williams et al. [52] Although animal models cannot fully reflect human IBS, the restraint stress murine model is considered suitable for representing the transient improvement in the stool consistency scores [53]. Previous studies have reported that restraint stress can induce visceral hyperalgesia by stimulation of colonic motility in response to rectal distension, resulting in increased fecal excretion in rats [52,54]. Moreover, restrained rats showed changes in the levels of corticotrophin-releasing factor (CRF), commonly considered a mediator in the gut-brain axis system [55,56], and low-grade mucosal inflammation with a significant increase in the mast cell and eosinophilic granulocyte counts [57]. In this respect, the restraint stress rat model is considered suitable for the evaluation of the effect of synbiotics before conducting studies involving humans. However, since the restraint model shows the only acute or sub-acute course of inflammation, a limitation of this study may be that we did not observe the characteristic conditions of IBS, such as chronicity persisting for at least 6 months. The main symptoms of IBS, abdominal pain, and pain relief with defecation, could not be assessed due to the inherent limitations associated with an animal study. Additional human studies are warranted to evaluate the efficacy of synbiotics for alleviating various IBS symptoms and patient-reported outcomes (PRO).

In conclusion, we report that a synbiotic containing *L. paracasei* and *O. humifusa* showed therapeutic effects in a murine IBS model and delineate the supporting mechanisms of action. This synbiotic may be a good therapeutic agent for IBS; therefore, its efficacy should be further assessed in a study involving humans.

## Supplementary Materials

The following are available online at https://www.mdpi.com/2072-6643/12/10/3205/s1, Table S1: Luminex Rat Magnetic Assay Kit information for measured cytokines, Table S2: Taxonomic relative abundance of bacterial taxa in feces of rats, Figure S1: Stool consistency score, Figure S2: Microbial community assay.
