# Supplementary material for: Effect of a Synbiotic Containing Lactobacillus paracasei and Opuntia humifusa on a Murine Model of Irritable Bowel Syndrome
Source: Nutrients. 2020 Oct 20;12(10):3205. doi: 10.3390/nu12103205 (PMC7594034; doi:10.3390/nu12103205)
Supplement: Supplementary file 1 [file nutrients-12-03205-s001.zip › nutrients-930770/supplementary figures.docx]

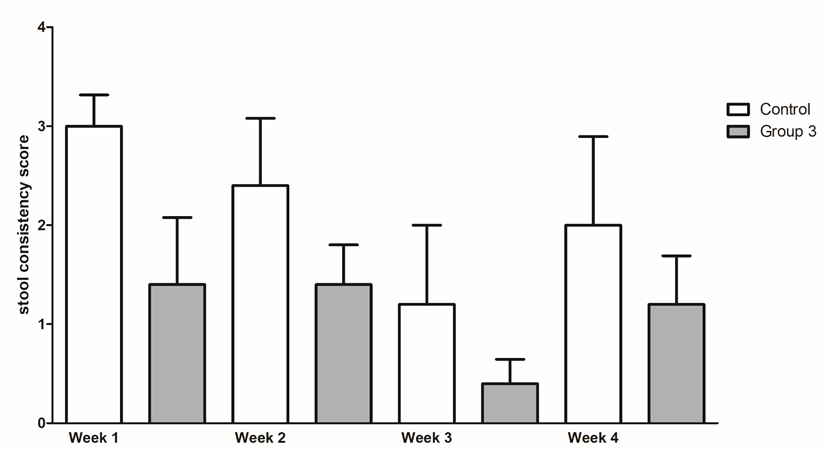


**Figure 1.** Stool consistency score. Results are expressed as the mean ± SEM (*n* $=$ 5/group). No significant difference was observed between the control group and treatment group 3.


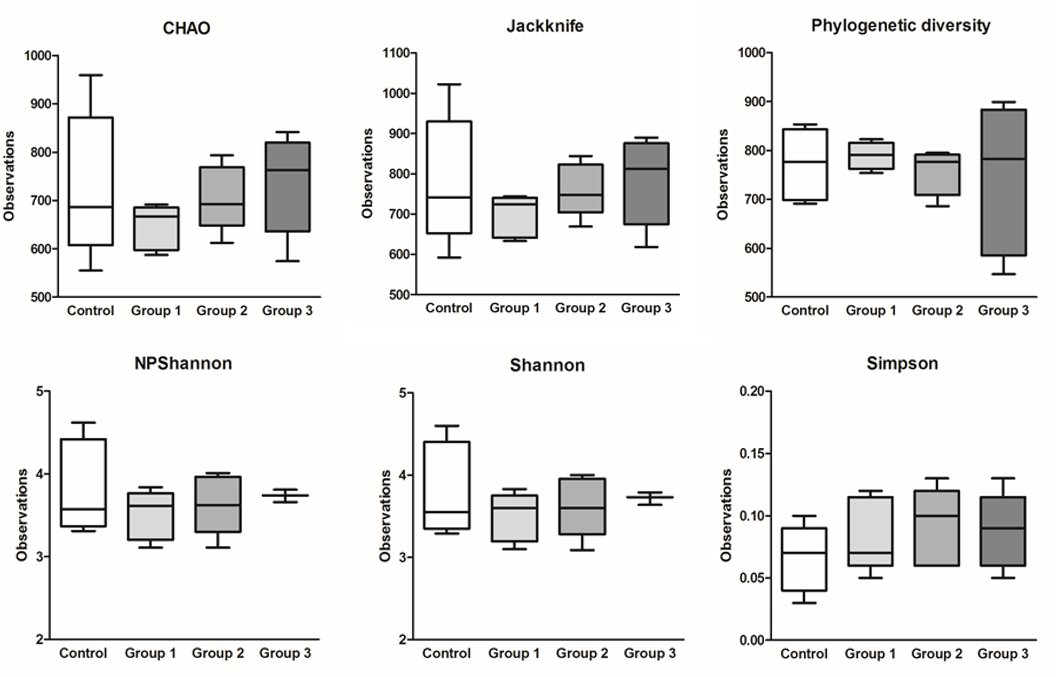


**Figure 2.** Microbial community assay. No significant difference was noted between the groups.
