# Supplementary material for: Effect of a Synbiotic Containing Lactobacillus paracasei and Opuntia humifusa on a Murine Model of Irritable Bowel Syndrome
Source: Nutrients. 2020 Oct 20;12(10):3205. doi: 10.3390/nu12103205 (PMC7594034; doi:10.3390/nu12103205)
Supplement: Supplementary file 1 [file nutrients-12-03205-s001.zip › nutrients-930770/Supple_Table 1.docx]

**Supplementary Table S1.** Luminex Rat Magnetic Assay Kit information for measured cytokines.

| **Luminex Rat Magnetic Assay (5-Plex) LXSARM-05** |  |  |  |  |  |  |
| --- | --- | --- | --- | --- | --- | --- |
| Analyte | BeadRegion | Standard Curve (pg/mL) | Serum | Plasma | CellCulture | Sensitivity (pg/mL) |
| INF-gamma | 18 | 509 - 123,600 | 1:02 | 1:02 | 1:02 | 70.9 |
| IL-1 beta/IL-1 F2 | 20 | 18.5 – 4,500 | 1:02 | 1:02 | 1:02 | 2.93 |
| IL-6 | 25 | 170 – 41,300 | 1:02 | 1:02 | 1:02 | 23.2 |
| IL-10 | 26 | 19.7 – 4,790 | 1:02 | 1:02 | 1:02 | 8.95 |
| TNF-alpha | 33 | 232 – 56,400 | 1:02 | 1:02 | 1:02 | 11.5 |

IFN, interferon; IL, interleukin; TNF, tumor necrosis factor
